# Random Walk Approximation for Stochastic Processes on Graphs

**DOI:** 10.3390/e25030394

**Published:** 2023-02-21

**Authors:** Stefano Polizzi, Tommaso Marzi, Tommaso Matteuzzi, Gastone Castellani, Armando Bazzani

**Affiliations:** 1 Department of Physics and Astronomy A. Righi, University of Bologna, 40127 Bologna, Italy; 2 Department of Physics and Astronomy, University of Florence, 50019 Sesto Fiorentino, Italy; 3 Department of Experimental, Diagnostic and Specialty Medicine, University of Bologna, 40138 Bologna, Italy

**Keywords:** non-linear processes, master equation, random walk, Fokker–Planck, bistability

## Abstract

We introduce the Random Walk Approximation (RWA), a new method to approximate the stationary solution of master equations describing stochastic processes taking place on graphs. Our approximation can be used for all processes governed by non-linear master equations without long-range interactions and with a conserved number of entities, which are typical in biological systems, such as gene regulatory or chemical reaction networks, where no exact solution exists. For linear systems, the RWA becomes the exact result obtained from the maximum entropy principle. The RWA allows having a simple analytical, even though approximated, form of the solution, which is global and easier to deal with than the standard System Size Expansion (SSE). Here, we give some theoretically sufficient conditions for the validity of the RWA and estimate the order of error calculated by the approximation with respect to the number of particles. We compare RWA with SSE for two examples, a toy model and the more realistic dual phosphorylation cycle, governed by the same underlying process. Both approximations are compared with the exact integration of the master equation, showing for the RWA good performances of the same order or better than the SSE, even in regions where sufficient conditions are not met.

## 1. Introduction

Stochastic processes are ubiquitous in nature and generate fluctuations that are not just noise, in particular in biochemical reactions occurring in single cells, where the copy number of the reactants can be relatively small, i.e., of the order of few hundreds [1,2]. At first glance, these molecules present in a small number were neglected and considered as unimportant, also because of the experimental difficulties to detect them. However, it soon became clear that in a biological system, all the reactions are coupled and even low copy numbers of, for instance, messenger RNA in bacteria [1] can determine the fate of the cell and result in peculiar properties due to large fluctuations, which can be very far from standard Poisson statistics [3]. Therefore, all stochastic properties became important to make predictions in that it was realized that noise can govern gene expression and in turn evolution [4,5], and it is necessary to adapt to new biological challenges [6]. Sometimes, fluctuations themselves can even generate phase transitions [7].

In these systems, it has thus been understood that considering only the macroscopic dynamics, neglecting then the stochasticity of the process, can be misleading and works only in very large systems. Therefore, a stochastic description, based on the master equation (ME), started being necessary [8]. Although in some simple cases, the ME can be solved analytically, mainly for linear processes, i.e., when the transition rates are constant, as for Brownian motion, most of the time, this is not possible. Those are, of course, cases of great interest, where self-organization phenomena occur, and the actual state of the system regulates its stability and evolution [9] and where the cell fate is determined by positive or negative feedback loops [10]. Moreover, nonlinearities are often at the origin of interesting phase transitions of biologic importance. Examples are gene expression networks [11], networks of chemical reactions showing non-equilibrium steady states and net fluxes, such as signal cellular pathways [12,13,14,15] and the phospho/dephosphorylation cycle of AMPA receptors in a single synaptic spine [16]. When the number of states of the system under study is finite, the process can be seen as a random walk on a graph. In this picture, a state of the system would be each possible observed form of it, quantified by some observables such as levels of proteins, messengers, organelles, or phenotypes. For instance, for gene expression networks, it would be the number of attractors (i.e., the number of cell types in the Waddington’s epigenetic landscape [11]) or the different levels of phosphorylation of a molecule in dual phosphorylation cycles [9] or again the number of chemicals involved in a signal transduction pathway. Each state of the system is then related to each other with some transition rates, generating a weighted graph, in which the weights associated to each edge are the transition rates and the nodes are the possible states of the system. This is a directed graph, since the pairs of nodes defining an edge are ordered by the direction of the transition; moreover, interesting behaviors, such as bifurcation phenomena, appear when the rates are not symmetric.

Recently, a lot of effort has been put into solving ME associated with non-linear systems on both developing accurate numerical techniques [17] and developing theoretical frameworks and approximation methods. Concerning the former, most of the numerical methods are derived from the progenitor of Monte Carlo sampling for the ME, the Gillespie algorithm [18], and the one exploiting the structure of the ME is remarkable [19]. On the other hand, the latter are mainly built on known results about the linear case, exploiting, for instance, the reaction velocities and then neglecting fast variables [20]. Theoretical methods to describe these systems at a mesoscopic scale can be divided into two main approaches: solving directly the ME by approximating the rates as constants or using the SSE, which transforms the ME in a diffusion equation by developing in terms of the system size [8,21], which is also known as van Kampen’s Ω-expansion.

Here, we present a new approach for giving an approximation of the solution of the ME exploiting the graph structure, the Random Walk Approximation (RWA), and we compare it with commonly used methods. Our method leverages both the two approaches mentioned above: we use the solution of the linear ME (the multinomial distribution), but we do not impose the transition rates to be constant, therefore hardly modifying the multinomial shape. This, as we will show, has two main advantages. First, we obtain a global solution and not only a local one, as it is with the SSE or with the pure multinomial approximation obtained approximating the transition rates as constant. The global solution is therefore able to approximate also the tail of the distribution and can contain itself the phase transition; if one exists, it allows indeed to establish whether a system undergoes a critical bifurcation point without further calculations. Second, it is much simpler and easier to use than the SSE, since all is needed is to solve an eigenvalue problem. Indeed, it gives an analytical solution that can be easily used for further calculations, such as, for instance, the computation of the entropy production of the process. Our method is general and can be applied to all systems governed by a ME, no matter the number of states, as long as the transition rates are given.

The paper is organized as follows. In Section 2, we introduce the RWA from a mathematical point of view and give some theoretical results on its performance. In Section 3, we briefly explain the numerical and analytical methods used as comparison with our RWA. In Section 4, we describe the common backbone of the models to which the approximation is applied and then give the explicit theoretical results for, first, a simple toy model considered to check the validity of the RWA and its essential properties, and, second, a more relevant model of biological importance, the dual phospho/dephosphorylation cycle (PdPC). Those models are chosen because analytical calculations were possible and for the ease of numerical implementation, which is useful to clarify how and to what extent the RWA can be successfully applied. In Section 5, we show the results of the application of the RWA to the described models and finally give a global discussion of our work.

## 2. The Random Walk Approximation

We introduce here the main topic of the paper, namely the Random Walk Approximation. In particular, among some theoretical results about its expected behavior, we state the sufficient conditions for it to be a reliable approximation in the sense of the $\ell^{1}$-norm.

### 2.1. Linear Master Equation for One Step Processes

We consider a generic system that is described by an ensemble of i=1,⋯,M states with transition rates $\pi_{ij}$ from the state j→i, which defines the probability that a “particle” (individual of the system) changes its state on a time unit. Therefore, one obtains a weighted graph (network) with *M* nodes and link weights given by the rates $\pi_{ij}$. If one considers the dynamics of *N* identical independent particles, the statistical properties of the system are described by the particle distribution function $\rho (\vec{n},t)$, which gives the probability of the network state $\vec{n}=\left(n_{1},\cdots ,n_{M}\right)$ where $n_{i}$ is the number of particles in the state *i* at time *t*. Since

$$|\vec{n}|=\sum_{i}n_{i}=N$$

is fixed, we are realizing a microcanonical ensemble (generalizations are possible by introducing an external node representing the reservoir coupled with the system). The evolution of the distribution function $\rho (\vec{n},t)$ is the master equation:

(1) $$\frac{\partial \rho }{\partial t}\left(\vec{n},t\right)=\frac{1}{N}\sum_{i,j}\left[\pi_{ij}E_{j}^{+}E_{i}^{-}n_{j}-\pi_{ji}n_{i}\right]\rho \left(\vec{n},t\right),$$

assuming the one-step process approximation (i.e., the probability that two particles move simultaneously in a time interval Δt is assumed to be o(Δt)). The one-step process is a continuous time Markov process, whose transition rates matrix allows only transitions between neighboring network states, which is not a strong experimental assumption, as long as you have a time resolution much higher than the typical transition time of a particle. The symbols $E_{i}^{\pm }$ denote the Van Kampen operators defined by:

$$E_{i}^{\pm }f\left(\vec{n}\right)=f\left(\vec{n}\pm {\hat{e}}_{i}\right)$$

for any function $f(\vec{n})$, where ${\hat{e}}_{i}$ is the standard canonical base of $Z^{M}$.

If the transition rates matrix $\pi_{ij}$ does not satisfy the detailed balance condition, the same is true for the ME (1); however, it is possible to prove that the stationary solution for the linear random walk is, regardless of whether detailed balance is verified or not, a maximum entropy distribution with the constraint that the average value of the particles in each node is finite (see Appendix A). In the following, detailed balance will not be assumed; therefore, we will deal, in general, with non-equilibrium processes. Moreover, in Equation (1), the distribution function $\rho (\vec{n})$ is extended to all possible states $\vec{n}\in Z^{M}$, defining $\rho (\vec{n},t)=0$ for all the non-physical states $|\vec{n}|\neq N$. Since the particles do not interact, it is also possible to analytically compute the solution $\rho (\vec{n},t)$ from the spectral properties of the Laplacian matrix [22] of the graph

(2) $$L_{ki}\left(\vec{n}\right)=\delta_{ki}d_{k}\left(\vec{n}\right)-\pi_{ki}\left(\vec{n}\right)\;d_{k}\left(\vec{n}\right)=\sum_{i}\pi_{ik}\left(\vec{n}\right),$$

where, for the linear case, the rates are independent of $\vec{n}$. In particular, the stationary solution is given by the multinomial distribution

(3) $$\rho \left(\vec{n}\right)=N!\prod_{i=1}^{M}\frac{p_{i}^{n_{i}}}{n_{i}!},$$

where the vector $\vec{p}=\left(p_{1},\ldots ,p_{M}\right)$ is the eigenvector corresponding to the null eigenvalue of *L* and the covariance matrix of the solution reads

(4) $$C_{ij}=Np_{i}\delta_{ij}-Np_{i}p_{j}.$$

We note that all the other eigenvalues of the Laplacian are positive and the second smallest eigenvalue is known as the Fiedler’s eigenvalue [22].

The multinomial solution is the same elegantly obtained by the application of the maximum entropy principle, as we show in Appendix A.

For N≫1, the multinomial converges to a symmetric distribution and the average values $\langle n_{i}\rangle =Np_{i}$ are also the mode of the multinomial distribution. The average dynamics can be directly computed from the ME

(5) $$\begin{array}{ccc} \langle {\dot{n}}_{k}\rangle & = & \sum_{|n|=N}n_{i}\frac{\partial \rho }{\partial t}\left(\vec{n},t\right)=\frac{1}{N}\sum_{i,j}\sum_{|n|=N}n_{k}\left[\pi_{ij}E_{j}^{+}E_{i}^{-}n_{j}-\pi_{ji}n_{i}\right]\rho \left(\vec{n},t\right)\\ & = & \sum_{i,j}\sum_{|n|=N}\left[\pi_{ij}E_{j}^{+}E_{i}^{-}\pi_{ij}\left(n_{k}-\delta_{jk}+\delta_{ik}\right)n_{j}-\pi_{ji}n_{k}n_{i}\right]\rho \left(\vec{n},t\right)\\ & = & \sum_{j}\left(\pi_{kj}\langle n_{j}\rangle -\pi_{jk}\langle n_{k}\rangle \right), \end{array}$$

whose critical points are the average values.

### 2.2. The Non-Linear Case

We consider now how the previous results generalize to non-linear random walks on graphs, when the transition probability rates depend on the network states $\pi_{ij}=\pi_{ij}\left(\vec{n}\right)$. These models allow to simulate the effect of particle interactions at the node, but a physical interpretation is needed to justify the one-step process assumption in the formulation of the ME. Indeed, each time a particle moves, the transition probabilities are instantaneously updated before another particle moves. Therefore, a synchronous evolution of the network in which many particles move at the same time gives rise to a different dynamical system. In the case $\pi_{ij}\left(\vec{n}\right)=\pi_{ij}\left(n_{i},n_{j}\right)$, the interactions are local (Markov random field) and the one-step process assumption is physically justified. The corresponding ME is:

(6) $$\frac{\partial \rho }{\partial t}\left(\vec{n},t\right)=\frac{1}{N}\sum_{i,j}\left[E_{j}^{+}E_{i}^{-}\pi_{ij}\left(\vec{n}\right)n_{j}-\pi_{ji}\left(\vec{n}\right)n_{i}\right]\rho \left(\vec{n},t\right).$$

We observe that the factor 1/N that defines the one-step process is a time scaling and scales the spectral properties of the Laplacian operator (defined hereafter), introducing a Fiedler’s eigenvalue of $O(N^{-1})$. Then, it is convenient to scale the time by a factor 1/N and remove this factor from the equation. Moreover, we assume $\pi_{ij}\left(\vec{n}\right)=\pi_{ij}\left(\vec{x}\right)$ where $x_{i}=n_{i}/N$, that is, the particle interactions depend on the density at each node *i*, in the limit N≫1. The stationary points of Equation (6) correspond to the eigenvectors $\vec{p}\left(\vec{x}\right)$ associated with the null eigenvalue of the Laplacian (2) (here in the density-dependent version) that satisfy the self-consistent condition:

(7) $$p_{i}^{*}\left({\vec{x}}^{*}\right)=x_{i}^{*}\;\mathrm{with}\;\sum_{i}x_{i}^{*}=1,$$

where ${\vec{x}}^{*}$ is the stationary solution of the deterministic dynamics associated with ME (6), $\sum_{i}L_{ji}\left(x_{i}^{*}\right)x_{i}^{*}=0$.

An explicit solution for the stationary distribution of the ME (6) is difficult due to the non-linear nature of the problem, except in the cases where the detailed balance holds. Indeed, detailed balance introduces the constraint of zero current between each pair of states, i.e., the process is at equilibrium, leading to a Maxwell–Boltzmann distribution with a potential energy depending on the state of the system (differently from the standard linear case) [9,23]. The potential energy can be computed by recursion from a function of the rates of the process, starting from an arbitrary value which will be uniquely determined by the normalization of ρ.

In order to build an approximate stationary solution, we introduce the eigenvector of components $p_{k}\left(\vec{x}\right)$ and consider the multinomial-like solution, which we refer to as the Random Walk Approximation of the stationary solution:

(8) $$\rho_{\vec{p}}^{*}\left(\vec{n}\right)=C\left(N\right)N!\prod_{k=1}^{M}\frac{p_{k}^{n_{k}}\left(\vec{x}\right)}{n_{k}!}\;x_{k}=n_{k}/N,$$

where C(N) is a normalizing factor. We are now interested in analyzing the behavior of the RWA with respect to the system size *N* and giving an estimate of the approximation error. First, in order to show that C(N) does not have a strong dependence on *N*, we use a perturbative approach by considering small perturbations of $\vec{p}\left(\vec{x}\right)$ around the stationary points: $p_{k}\left(\vec{x}\right)=p_{k}^{*}+\Delta p_{k}\left(\vec{x}\right)$, where the perturbations $\Delta p_{k}$ satisfy the condition $\sum_{k}\Delta p_{k}=0$. Then, by injecting this into (8), we show that C(N)=O(1) for N≫1, with a weak dependence on *N* (see detailed calculation in Appendix B).

We now give some conditions on the validity of Equation (7) for the critical points. For N≫1, we compute the modes of the distribution (8) from the condition

$$\log\frac{n_{i}}{Np_{i}}-\sum_{k}\frac{\partial p_{k}}{\partial x_{i}}\frac{n_{k}}{Np_{k}}≃0$$

If one introduces the $z_{k}=n_{k}/\left(Np_{k}\right)=x_{k}/p_{k}$, the relation can be written in the form

(9) $$\log z_{i}=\sum_{k}\frac{\partial p_{k}}{\partial x_{i}}z_{k},$$

which is clearly satisfied for $z_{k}=1$, since:

$$\sum_{k}\frac{\partial p_{k}\left(\vec{x}\right)}{\partial x_{i}}=0.$$

Therefore, as long as the matrix ∂p/∂x (i.e., the transpose of the Jacobian matrix of the eigenvector $\vec{p}\left(\vec{x}\right)$) has all the eigenvalues λ<1, the self-consistent average solution $x_{i}^{*}=p_{i}^{*}\left({\vec{x}}^{*}\right)$ (the unperturbed solution for $\Delta p_{k}=0$) is the only critical point of the distribution (8) and the distribution is peaked at the critical point with a spread $O(\sqrt{N})$. This is because one cannot have a tangency condition between $\log z_{i}$ and the right-hand side of Equation (9) in such a way that self-consistency is verified. When we have an eigenvalue λ≥1, the perturbation $\Delta p_{k}$ may introduce other solutions to Equation (9) that are critical points for the RWA distribution but not for the stationary distribution of Equation (6). We remark that this condition is also the condition necessary for a bifurcation phenomenon, i.e., when the distribution (8) becomes bi-(or multi-)modal. Indeed, if one considers the self-consistent Equation (7) for the average dynamics, the existence of a bifurcation is equivalent to the existence of a null eigenvalue for the matrix

$$\delta_{ik}-\frac{\partial p_{i}}{\partial x_{k}},$$

computed at the critical point. In other words, when a bifurcation phenomenon occurs for the self-consistent critical points of the average dynamics (7), the RWA (8) may have spurious stationary points that do not correspond to those of the average dynamics. Then, in this case, it is not guaranteed that the RWA is a good global approximation of the ME stationary solution.

We are now ready to compute the error of the approximation of the RWA (8) and its scaling with *N*. We will show that it depends on the derivative of the probabilities $p_{i}\left(\vec{x}\right)$. The intuition behind this is that fluctuations should be small with respect to the inhomogeneity of the system, which is represented by the derivatives of the $p_{i}\left(\vec{x}\right)$. An estimate of the error $|\rho_{\vec{p}}^{*}\left(\vec{n}\right)-\rho^{s}\left(\vec{n}\right)|,$ where $\rho^{s}$ is the exact stationary distribution, can be achieved by substituting the distribution (8) in the ME:

$$\ell^{1}\text{-error}=\sum_{i,j}\left[E_{j}^{+}E_{i}^{-}\pi_{ij}\left(\vec{x}\right)n_{j}-\pi_{ji}\left(\vec{x}\right)n_{i}\right]\rho_{\vec{p}}^{*}\left(\vec{n}\right)$$

When N≫1, the main contribution to the error is due to the dependence of $p_{k}$ from the densities $\vec{x}$:

(10) $$\ell^{1}\text{-error}≃\frac{1}{N}\sum_{i,j}\pi_{ij}\left(\vec{x}\right)n_{j}\sum_{k}\left(\frac{\partial p_{k}}{\partial x_{j}}-\frac{\partial p_{k}}{\partial x_{i}}\right)\frac{n_{k}}{p_{k}}\rho_{\vec{p}}^{*}\left(\vec{n}\right).$$

Considering the fluctuations around the critical point of order $\Delta n_{i}=p_{i}O\left(\sqrt{N}\right)$ in the limit N≫1, the largest contribution to Equation (10) is proved to be of order $O\left(∥\frac{\partial p}{\partial x}∥\right)$ (details in Appendix C). Finally, we can write the estimate for the $\ell^{1}$-error:

(11) $${∥\rho_{\vec{p}}^{*}\left(\vec{n}\right)-\rho^{s}\left(\vec{n}\right)∥}_{1}=O\left(∥\frac{\partial p}{\partial x}∥\right),$$

this means that the error does not depend on *N*, since the rates depend only on the densities $\vec{x}$ and not on *N*. The error is then independent of *N* if the distribution reaches its peak at the critical values with a spread $O(\sqrt{N})$. At a bifurcation of the critical point, when a bimodal distribution is expected, the previous estimate (11) could no longer be valid for both the increased spread of the peak and for the existence of a very small Fiedler’s eigenvalue for the matrix $L_{ij}\left(\vec{x}\right)$.

## 3. Methods

As stated previously, except for systems in which the detailed balance condition holds, it is a challenging task to compute the stationary distribution because of the non-linearity of the problem. In this section, a standard method that will be applied in the following, the System Size Expansion (SSE) is presented and adapted to the situation of interest. The exact solution of the ME is given by the numerical integration of the ME, obtained with the Runge–Kutta (RK) algorithm [24,25] (RK5(4) or Runge–Kutta–Dormand–Prince, Python 3.10.4, mainly from module scipy v1.9.1), in an adapted way for one-step processes, which fastens the numerical convergence [26]. Details are given in Appendix D, and the code for all the results on the dual PdPC used in this paper is publicly available on GitHub [27].

### 3.1. System Size Expansion

The SSE considers the thermodynamic limit N→∞ of the master Equation (1), or (6) for non-linear processes, in the continuous variables $\vec{x}=\vec{n}/N$. Developing the ME up to terms of order $O(N^{-2})$ one obtains the Fokker–Planck (FP) equation associated to the ME. If for linear cases, the SSE is straightforward [8], the calculations for the non-linear case are a little more cumbersome. The details of the calculations for both cases are given in Appendix E. In both cases, the SSE locally approximates the multinomial distribution (8) as N→∞ by a Gaussian distribution in the neighborhood $\delta \vec{x}=\vec{x}-{\vec{x}}^{*}$ of the critical point ${\vec{x}}^{*}$. The general solution for the non-linear case is the Gaussian:

(12) $$\rho_{SSE}^{*}\left(\delta \vec{x}\right)=\frac{1}{{\left(2\pi \right)}^{3/2}\;{\left|\Sigma \right|}^{1/2}}\exp\left[-\frac{1}{2}\delta {\vec{x}}^{T}\Sigma^{-1}\delta \vec{x}\right],$$

where the covariance matrix Σ solves the continuous Lyapunov equation [8]:

(13) $$-\bar{A}\Sigma -\Sigma {\bar{A}}^{T}+D^{*}=0$$

in which we denoted as $\bar{A}$ the matrix such that:

(14) $${\bar{A}}_{ij}=\sum_{k}\left[L_{ik}^{*}\delta_{jk}+\frac{\partial L_{ik}}{\partial x_{j}^{*}}x_{k}^{*}\right],$$

where $L_{ik}^{*}$ is the Laplacian at the critical point, and with a slight abuse of notation, we denoted $\frac{\partial L_{ik}}{\partial x_{j}^{*}}$ as the derivative of the Laplacian calculated at the critical point. The positive symmetric matrix $D^{*}$ is the diffusion matrix:

$$D_{ij}\left(\vec{x}\right)=\frac{1}{N}\left[\sum_{k}\delta_{ij}\left(\pi_{ik}\left(\vec{x}\right)x_{k}+\pi_{ki}\left(\vec{x}\right)x_{i}\right)-\left(\pi_{ij}\left(\vec{x}\right)x_{j}+\pi_{ji}\left(\vec{x}\right)x_{i}\right)\right],$$

calculated as well at the critical point. In the non-linear case, these equations can only be solved numerically.

## 4. Model

**Figure 1:**
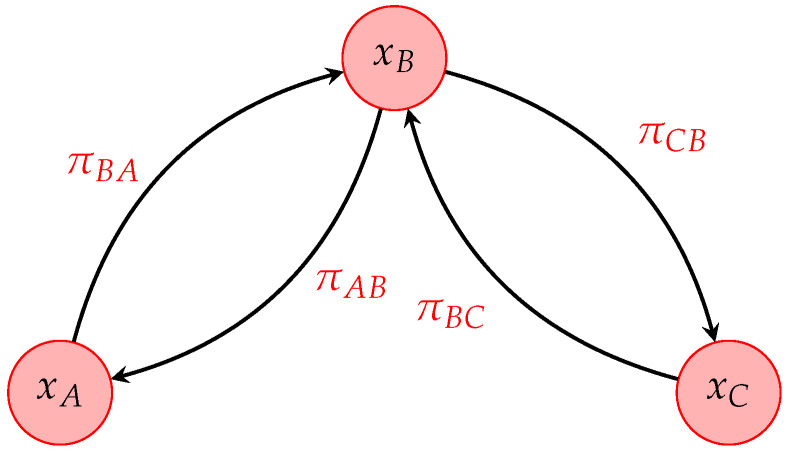
Scheme of the three-state model.

The general setup we chose to test the RWA consists of a three-state (chemical species) process ruled by two underlying reaction cycles; a general scheme is reported in Figure 1. This is a typical setup for biochemical reactions in single cells, and it is a still reasonably simple setup to apply the RWA.

The state of the system is represented by a three-dimensional vector $\vec{n}=\left(n_{A},n_{B},n_{C}\right),$ and it can be reduced to two dimensions $\vec{n}=\left(n_{A},N-n_{A}-n_{C},n_{C}\right)$ by assuming the conservation on the total number of particles $|\vec{n}|=N$. The dependence of the dynamics with respect to $\vec{n}$ can be removed by defining the concentration vector $\vec{x}=\vec{n}/N$. The transition rates $\pi_{ij}$ from a species *j* to a species *i* may depend on this vector, and they allow to build a parameterized transition matrix $\Pi (\vec{x})$ and Laplacian matrix $L(\vec{x})$.

In order to analyze the time evolution of the system, we write down explicitly the deterministic equation of the dynamics for the density (or concentration) vector:

(15) $$\left\{\begin{array}{c} \frac{dx_{A}}{dt}=\pi_{AB}\left(\vec{x}\right)x_{B}-\pi_{BA}\left(\vec{x}\right)x_{A}\\ \frac{dx_{C}}{dt}=\pi_{CB}\left(\vec{x}\right)x_{B}-\pi_{BC}\left(\vec{x}\right)x_{C} \end{array}\right.$$

Notice that this system of equations is equivalent to the system associated to the dynamics of the graph obtainable through the Laplacian matrix:

(16) $$\frac{d\vec{x}}{dt}=-L\left(\vec{x}\right)\vec{x}$$

in which the equation associated to the time derivative of $x_{B}$ is redundant as a consequence of the constraint on the concentrations $|\vec{x}|=1$. The critical states of the system, which can be stable or unstable, correspond to the vectors ${\vec{x}}^{*}$ with a null time derivative in (15): this is equivalent to considering the macroscopic dynamics. At the base of the RWA, there is the eigenvector with null eigenvalue of the Laplacian $L(\vec{x})$, that for our three-state model depends on the current $\vec{x}$ by:

$$\begin{array}{cc} p_{A}\left(\vec{x}\right) & =\frac{\pi_{AB}\left(\vec{x}\right)\pi_{BC}\left(\vec{x}\right)}{\pi_{AB}\left(\vec{x}\right)\pi_{BC}\left(\vec{x}\right)+\pi_{CB}\left(\vec{x}\right)\pi_{BA}\left(\vec{x}\right)+\pi_{BA}\left(\vec{x}\right)\pi_{BC}\left(\vec{x}\right)}\\ p_{C}\left(\vec{x}\right) & =\frac{\pi_{CB}\left(\vec{x}\right)\pi_{BA}\left(\vec{x}\right)}{\pi_{AB}\left(\vec{x}\right)\pi_{BC}\left(\vec{x}\right)+\pi_{CB}\left(\vec{x}\right)\pi_{BA}\left(\vec{x}\right)+\pi_{BA}\left(\vec{x}\right)\pi_{BC}\left(\vec{x}\right)} \end{array}$$

with the self-consistent condition (7).

If we want instead to consider the stochasticity of the process, the time evolution of the probability distribution $\rho (\vec{x},t)$ associated to the states of the system is constructed by taking into account all of the possible exchanges of the particles, as in (6). Explicitly, the ME reads:

(17) $$\begin{array}{cc} \frac{\partial \rho (x_{A},x_{C},t)}{\partial t}= & \pi_{AB}\left(x_{A}-\frac{1}{N},x_{C}\right)\left(1-x_{A}+\frac{1}{N}-x_{C}\right)\rho \left(x_{A}-\frac{1}{N},x_{C},t\right)+\\ \; & -\pi_{AB}\left(x_{A},x_{C}\right)\left(1-x_{A}-x_{C}\right)\rho \left(x_{A},x_{C},t\right)\\ \; & +\pi_{BA}\left(x_{A}+\frac{1}{N},x_{C}\right)\left(x_{A}+\frac{1}{N}\right)\rho \left(x_{A}+\frac{1}{N},x_{C},t\right)+\\ \; & -\pi_{BA}\left(x_{A},x_{C}\right)x_{A}\rho \left(x_{A},x_{C},t\right)\\ \; & +\pi_{CB}\left(x_{A},x_{C}-\frac{1}{N}\right)\left(1-x_{A}-x_{C}+\frac{1}{N}\right)\rho \left(x_{A},x_{C}-\frac{1}{N},t\right)+\\ \; & -\pi_{CB}\left(x_{A},x_{C}\right)\left(1-x_{A}-x_{C}\right)\rho \left(x_{A},x_{C},t\right)\\ \; & +\pi_{BC}\left(x_{A},x_{C}+\frac{1}{N}\right)\left(x_{C}+\frac{1}{N}\right)\rho \left(x_{A},x_{C}+\frac{1}{N},t\right)+\\ \; & -\pi_{BC}\left(x_{A},x_{C}\right)x_{C}\rho \left(x_{A},x_{C},t\right), \end{array}$$

where we explicitly removed the dependence on $x_{B}$. Therefore, while the macroscopic approach provides an average dynamics of the system in the N→∞ limit, the ME describes statistically the time evolution of the probability distribution together with its stationary properties. In the thermodynamics limit N→∞, fluctuations are negligible, and the stationary state of the ME approach recovers the macroscopic kinetics, since the distribution converges toward a delta function peaked on the critical point of the average dynamics. If the system satisfies the detailed balance condition, the stationary solution coincides with the equilibrium solution and it can be computed in a closed form corresponding to a Maxwell–Boltzmann distribution [28]. However, in non-equilibrium steady states, an explicit solution of the stationary distribution cannot be obtained as a consequence of the effect of stationary currents.

According to the functional form of the transition matrix $\Pi (\vec{x})$, we consider two different models that follow the scheme in Figure 1: a toy model and a biologically-inspired model, i.e., the dual PdPC.

### 4.1. Toy Model

As a consequence of their complex dynamics, biologically inspired models often depend on a large set of parameters, and the sensitivity on this high-dimensional parameters space can make it difficult to carry out a systematic study of the model itself. Therefore, before generalizing to a more reliable and known model such as the dual PdPC, we perform the analysis on a simple model depending on a one-dimensional parameter space. In particular, referring to Figure 1, the rates are:

$$\begin{array}{cc} \pi_{AB} & =1\;\pi_{BA}\left(\vec{x}\right)=\theta \left(x_{A}\right)\\ \pi_{CB} & =1\;\pi_{BC}\left(\vec{x}\right)=\theta \left(x_{C}\right) \end{array}$$

in which θ(z) is a threshold function depending on the unique control parameter α in the form:

(18) $$\theta \left(x\right)=1-\alpha +\alpha {\left(1-x\right)}^{2},\;\alpha \in \left[0,1\right].$$

When α=0, the system is linear, and all the transitions are equally likely, whereas on the opposite, when α=1, a large fraction of particles in states *A* and *C* drops the transition rates toward state *B*, leading to bistability. This model can be interpreted as a Markov process on a graph whose time evolution is governed by Equation (16). It is possible to compute the critical condition for the bifurcation of the symmetric equilibrium $x_{1}=x_{3}$ for the average self-consistent Equation (7):

(19) $$f\left(x\right)=1+\frac{d\theta }{dx}\frac{1}{\theta (x)(2+\theta (x))}=0,$$

of which a solution can be computed numerically and is for 0.8<α<0.9.

### 4.2. Dual Phospho/Dephosphorylation Cycles

We analyze here the dual phospho/dephosphorylation cycles. In order to analyze the time evolution of the system, we make use of the Michaelis–Menten (MM) approach, which provides an average description of the dynamics of the enzyme kinetics under the hypothesis of the quasi-steady-state approximation. In particular, the latter consists of assuming a constant concentration for the enzyme–substrate complex and results in rates being a non-linear function of the system state. The components of the concentration vector $x_{A}$, $x_{B}$ and $x_{C}$ represent, respectively, the unphosphorylated, phosphorylated and double-phosphorylated substrates. Each of the four reactions of the dual PdPC follows the same MM enzyme kinetic scheme, which consists of a reversible and an irreversible process. In particular, the former involves a substrate *S* which binds to an enzyme *E*, forming an enzyme–substrate complex ES. The latter uses this complex to produce a product *P* and regenerates the free enzyme *E*.

The whole system of reactions reads:

$$\begin{array}{c} \begin{array}{c} x_{A}+E_{1}\overset{k_{f1}}{\underset{k_{b1}}{⇌}}E_{1}x_{A}\overset{k_{c1}}{\to }x_{B}+E_{1} \end{array}\\ \begin{array}{c} x_{B}+E_{1}\overset{k_{f2}}{\underset{k_{b2}}{⇌}}E_{1}x_{B}\overset{k_{c2}}{\to }x_{C}+E_{1} \end{array}\\ \begin{array}{c} x_{B}+E_{2}\overset{k_{f3}}{\underset{k_{b3}}{⇌}}E_{2}x_{B}\overset{k_{c3}}{\to }x_{A}+E_{1} \end{array}\\ \begin{array}{c} x_{C}+E_{2}\overset{k_{f4}}{\underset{k_{b4}}{⇌}}E_{2}x_{C}\overset{k_{c4}}{\to }x_{B}+E_{2} \end{array} \end{array}$$

in which for each reaction *i*, the kinetic constants $k_{fi}$, $k_{bi}$ and $k_{ci}$ represent the forward, backward and catalytic constants, respectively. Under the standard quasi-steady-state assumption (sQSSA) for the enzyme–substrate complex concentration, the transition rates assume the following form [29]:

$$\begin{array}{cc} \pi_{AB}\left(\vec{x}\right) & =\frac{k_{4}v_{2}}{k_{4}k_{2}+k_{4}x_{B}+k_{2}x_{C}}\;\pi_{BA}\left(\vec{x}\right)=\frac{k_{3}v_{1}}{k_{1}k_{3}+k_{1}x_{B}+k_{3}x_{A}}\\ \pi_{CB}\left(\vec{x}\right) & =\frac{k_{1}v_{3}}{k_{1}k_{3}+k_{1}x_{B}+k_{3}x_{A}}\;\pi_{BC}\left(\vec{x}\right)=\frac{k_{2}v_{4}}{k_{2}k_{4}+k_{4}x_{B}+k_{2}x_{C}} \end{array}$$

in which $k_{i}=\left(k_{bi}+k_{ci}\right)/k_{fi}$ is the MM constant and $v_{i}$ represents the maximal rate velocity associated to reaction *i*.

We note that the literature proposes alternative methods to study the dynamics of the dual PdPC, different from the sQSSA [30], and our RWA can be applied to any kind of chosen reaction kinetics, since all of them depend on densities. However, for the purpose of this paper, which is to apply the RWA to a real case, we selected the sQSSA.

The complexity of the model is reduced by assuming $k_{1}=k_{4}=0.1$, $k_{2}=k_{3}=1$, $v_{1}=v_{4}=1$ and $v_{2}=v_{3}$. Therefore, $v_{2}$ is the control parameter which governs the bifurcation phenomena, which, with the given parameters occurs at $v_{2}=2.5$. By computing the critical states, one obtains a solution ${\vec{x}}_{1}^{*}$ of the form:

(20) $${\vec{x}}_{1}^{*}=\left(\frac{k_{1}v_{2}}{k_{2}v_{1}+2k_{1}v_{2}},\frac{k_{2}v_{1}}{k_{2}v_{1}+2k_{1}v_{2}},\frac{k_{1}v_{2}}{k_{2}v_{1}+2k_{1}v_{2}}\right)$$

which exists independently of the value $v_{2}$. Notice that this solution automatically satisfies the constraints on the density $0\leq x_{1,(A,B,C)}^{*}\leq 1$, $|{\vec{x}}_{1}^{*}|=1$. Moreover, if the following conditions on $v_{2}$, which come from the existence of a stationary real solution of Equation (15) and the requirements for ${\vec{x}}^{*}$ to be a probability distribution, are satisfied simultaneously:

(21) $$\begin{array}{cc} {\left[v_{2}-v_{1}\left(1+k_{2}\right)\right]}^{2} & \geq {\left[2k_{1}v_{2}\right]}^{2}\\ v_{2} & \geq v_{1}\\ v_{2} & \geq v_{1}\left(1+k_{2}\right), \end{array}$$

we have two additional and symmetric critical states in the form:

(22) $$\begin{array}{c} {\vec{x}}_{2}^{*}=\left(x_{+}^{s},\frac{k_{2}v_{1}}{v_{2}-v_{1}},x_{-}^{s}\right) \end{array}$$

(23) $$\begin{array}{c} {\vec{x}}_{3}^{*}=\left(x_{-}^{s},\frac{k_{2}v_{1}}{v_{2}-v_{1}},x_{+}^{s}\right) \end{array}$$

in which $x_{\pm }^{s}$ are the roots of the following second-order equation:

$${\left(x_{\pm }^{s}\right)}^{2}-\frac{x_{\pm }^{s}}{v_{2}-v_{1}}\left(v_{2}-v_{1}\left(1+k_{2}\right)\right)+{\left(\frac{k_{1}v_{2}}{v_{2}-v_{1}}\right)}^{2}=0$$

Therefore, if the conditions in Equation (21) are met, then we have a bistable system, with two stable solutions ${\vec{x}}_{2}^{*}$, ${\vec{x}}_{3}^{*}$ and an unstable critical point ${\vec{x}}_{1}^{*}$. Otherwise, the latter is the unique stable point, and the system is monostable (with the chosen parameters, this occurs when $v_{2}<2.5$).

## 5. Results

We describe here the comparison between the RWA and the theoretical relations from Section 2 or the other approximation methods commonly used in the literature. In particular, we use the toy model to observe in action the discussion on RWA of Section 2 and the dual PdPC to prove its utility in a typical research situation compared to currently used methods.

### 5.1. Toy Model

**Figure 2:**
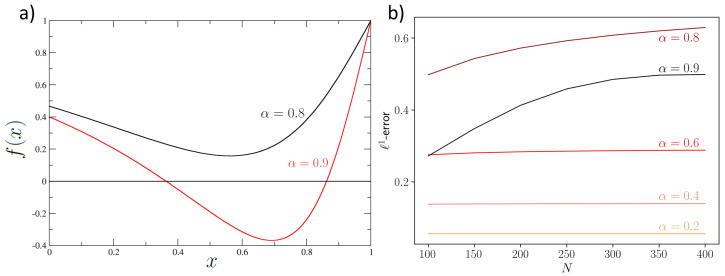
(**a**) Plot of the critical condition (19) for α=0.8 (black line) and α=0.9 (red line) for which the intersection with the *x*-axis is clearly visible, showing a bifurcation. (**b**) $\ell^{1}$-error for the RWA of the ME associated to Equation (15) using the toy model transition rates for different values of α. The error is *N*-independent before the bifurcation, whereas it increases with *N* near the bifurcation.

A direct calculation gives $\partial p_{i}/\partial x_{j}\propto g(\alpha )$, where *g* is a function independent of *N*, monotonically increasing with respect to α (see Appendix F), which is the control parameter. We can check the validity of the estimate (11) by computing the RWA $\ell^{1}$-error with respect to the numerical solution of the ME associated to Equation (15). The results are plotted in Figure 2 (right) for different values of the parameter α. We observe that for values α≤0.6, the $\ell^{1}$-error of the RWA is independent of the particle number, and it is proportional to the α value. When α=0.8, we are near the bifurcation condition, and the error becomes sensitive to the particle number, increasing with the number of particles, since the perturbation approach starts being less accurate. However, at α=0.9, when the distribution $\rho (\vec{n})$ is bimodal (Figure 2), the existence of local peaks may reduce the $\ell^{1}$-error, but one has no warranty that spurious critical points exist (i.e., critical points for the distribution that are not solutions of (7)).

In Appendix F, we show that the error on the variance is also independent of *N* and increases quickly close to bifurcation, starting having the same dependence on *N* as the $\ell^{1}$-error.

**Figure A1:**
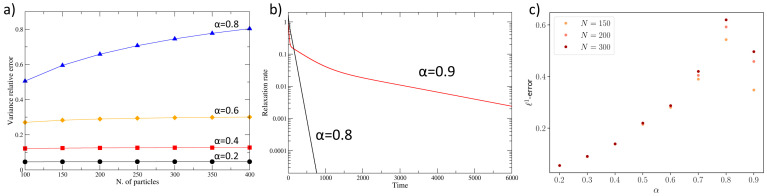
(**a**) Plot of the relative variance error of the RWA for different values of the parameter α. We observe that the error values correspond to the $\ell^{1}$-errors shown in Figure 2; (**b**) relaxation rate in log-scale of the numerical solution of the master equation associated to (15) for α=0.4 (before the bifurcation) and α=0.9 (after the bifurcation with N=200 particles; (**c**) plot of the $\ell^{1}$-error as a function of α for different values of N=150,200,300. Far from the bifurcation, the curve is monotonically increasing (approximately linear for small α), while when approaching the bifurcation, changes in the monotony of the function emerge.

Finally, at the bifurcation point, we also observe a change in the spectral properties of the Laplacian: this is shown in Figure A1c, where we plot the relaxation rate of the numerical solution of the ME associated to Equation (15) for the parameter values α=0.4 (before the bifurcation) and α=0.9 (after the bifurcation) at N=200 particles.

The change in the relaxation process depends on the Fiedler’s eigenvalue, as we discussed in Section 2: before the bifurcation, we have an explicit exponential relaxation dominated by the Fiedler’s eigenvalue that is far from zero; after the bifurcation, when the distribution is bimodal, the relaxation process shows different exponential slopes because the Fiedler’s eigenvalue (and possibly also other successive eigenvalues) is very close to zero, so the relaxation time is much longer.

### 5.2. Dual Phospho/Dephosphorylation Cycles

**Figure 3:**
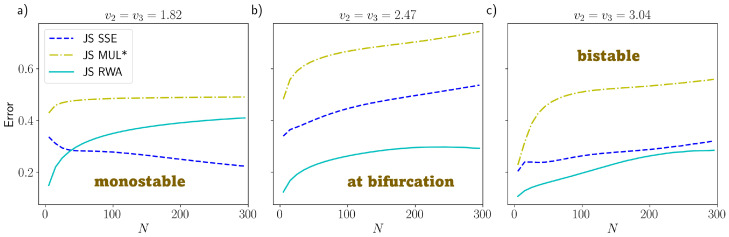
Jensen–Shannon (JS) error for the dual PdPC model with respect to the Runge–Kutta numerically integrated distribution vs. the number of particles *N*. The colors refer to: System Size Expansion (dashed blues), multinomial approximation (dashed-dotted yellow, referred to as MUL${}^{*}$) and Random Walk Approximation (solid cyan): (**a**) Monostable state with control parameter $v_{2}=1.82$. (**b**) Close to criticality, with $v_{2}=2.47$. (**c**) Bistable with $v_{2}=3.04$. The other parameters are set to $k_{1}=0.1,k_{2}=1$ for all plots.

The dual PdPC show a more complex behavior that is more similar to what one can encounter in real case scenarios. This is the reason why we focus here on the comparison between the RWA and other common approximation methods usually applied in the literature. Those alternatives are: the SSE, probably the most used, and the standard multinomial solution of the ME, which is obtained by linearizing the ME and considering only the zero-order expansion of the rates around the critical point ${\vec{x}}^{*}$ and then using directly the linear solution (3). In Figure 3, we plot the error of each approximation method with respect to the direct numerical integration of the ME through the RK4(5) algorithm (Section 3). Here, we decided to use as error metric the Jensen–Shannon (JS) divergence [31,32]. It is a measure based on the entropy of the distribution, and it is symmetric and normalized to 1 (with $\log_{2}$), giving then an absolute scale of comparison between the information content of the different approximations. The same plot, but with the $\ell^{1}$-norm, is given in Appendix F, showing the same scaling with respect to *N*, but with the drawback that is less accurate for the comparison, because it is more sensitive to the discretization of the space and it is not absolute. We note that our theoretical results of Section 2 are rigorous only with respect to the $\ell_{1}$-error. Nonetheless, since the JS divergence is based on the product of a probability distribution with the logarithm of a ratio between probabilities (which is of order 1 in *N*), and it does not involve exponentiation of ρ, it is expected to behave with *N* in the same way as the $\ell^{1}$-error.

**Figure 4:**
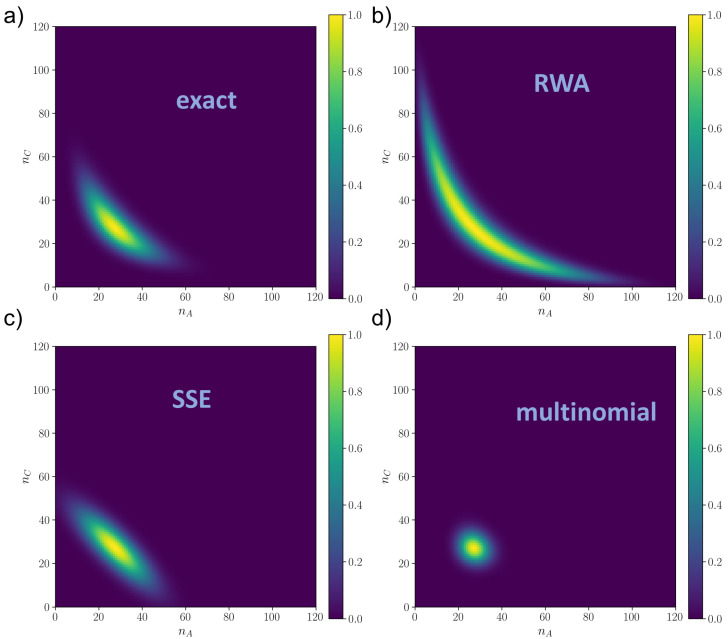
Rescaled distributions $\rho \left(n_{A},n_{C}\right)/\rho_{max}$ in the monostable regime ($v_{2}=1.82$) as resulted from (**a**) numerical integration of the master equation via Runge–Kutta method ($\rho_{max}=2.48\times 10^{-3}$); (**b**) Random Walk Approximation ($\rho_{max}=1.14\times 10^{-3}$); (**c**) System Size Expansion ($\rho_{max}=2.47\times 10^{-3}$) and (**d**) linear approximation of the master equation (multinomial distribution) ($\rho_{max}=6.78\times 10^{-3}$). N=205, $k_{1}=0.1,k_{2}=1$ for all plots.

**Figure 5:**
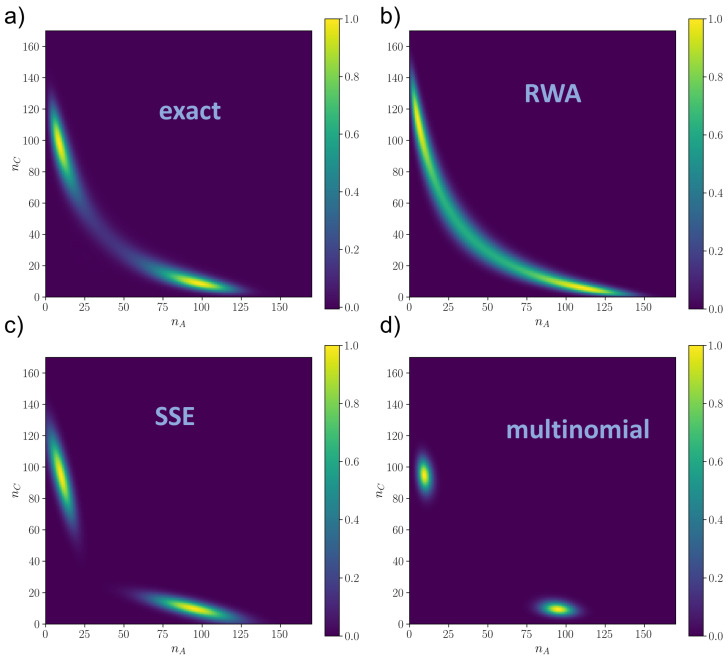
Rescaled distributions $\rho \left(n_{A},n_{C}\right)/\rho_{max}$ in the bistable regime ($v_{2}=3.04$) as resulted from (**a**) numerical integration of the master equation via Runge–Kutta method ($\rho_{max}=1.22\times 10^{-3}$); (**b**) Random Walk Approximation ($\rho_{max}=7.39\times 10^{-4}$); (**c**) System Size Expansion ($\rho_{max}=1.42\times 10^{-3}$) and (**d**) linear approximation of the Master Equation (multinomial distribution) ($\rho_{max}=3.68\times 10^{-3}$). N=205, $k_{1}=0.1,k_{2}=1$ for all plots.

Our results show two main things. First of all, as in Figure 2, the error of the RWA (both for the JS and for the $\ell^{1}$-norm, as shown in Appendix F) does not depend much on *N*, except at very low *N*, where stochastic fluctuations are important. This confirms the analytical result (10), because, by direct calculation, the derivatives $\frac{\partial p_{i}}{\partial x_{j}}$ are, in this model also independent of *N*, even if it does not have a simple proportionality with the control parameter $v_{2}$. Close to the bifurcation or after the bifurcation (when the system is bistable, plots (**b**) and (**c**) of Figure 3), some spurious dependence on *N* seems to appear, but especially for the JS measure, the error is still reasonably constant with *N*, suggesting that the RWA can be a good approximation even during and after the bifurcation. Second, the RWA, in addition to always performing significantly better than the standard multinomial approximation, is comparable to or better than the SSE, again even close to or after the bifurcation. Together, these numerical results suggest that the RWA can be reliable even when the mathematical conditions given in Section 2 are not met, and it is likely more general that what we proved. In this respect, we should note that after or close to the bifurcation, even the SSE has some issues of applicability. Indeed, the SSE (whose distribution is shown in Figure 4 for the monostable regime and Figure 5 for the bistable regime) in this case is constructed artificially, centering the two symmetric Gaussian distributions on the critical points ${\vec{x}}_{2}^{*}$ and ${\vec{x}}_{3}^{*}$. This works well for the parameters of Figure 5, but in general, when the two Gaussians are partially overlapped, this can lead to incorrect distributions. The issue would be even more problematic in systems with more than two stable states. This is a consequence of the fact that the SSE is a local approximation that only works in a neighborhood of ${\vec{x}}^{*}$, while the RWA is global.

In order to give some measures of the error on the whole approximated distribution, we can state that at the mesoscale for N=200 and $v_{2}=1.82$, the RWA makes a JS error of about 32%, while the SSE is 25%; at $v_{2}=2.47$, close to bifurcation, the RWA has an error of 29%, while the SSE is 50% and at $v_{2}=3.04$, in the bistable regime, the RWA has an error of 27%, while the SSE is 29%. These errors could seem high in general, but it has to be considered that these are errors on the information contained in the whole distribution, even considering the tails, which are usually neglected.

**Figure A3:**
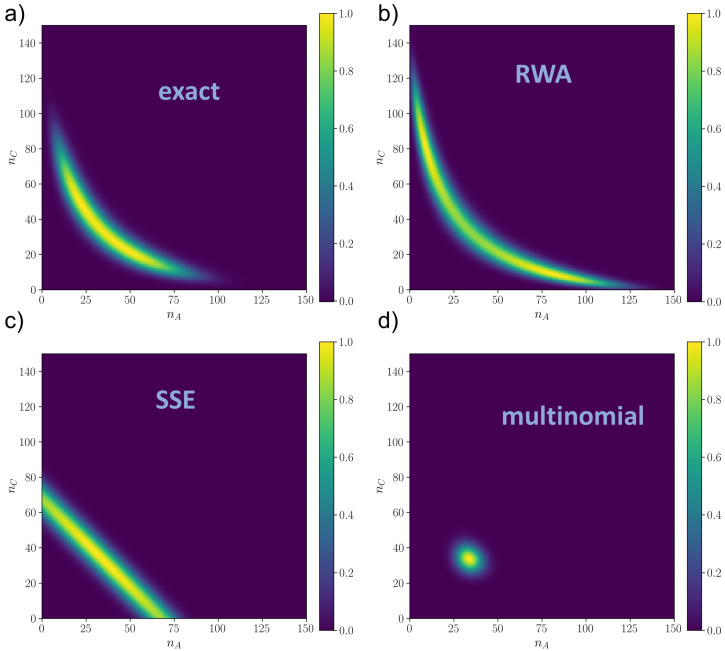
Rescaled distributions $\rho \left(n_{A},n_{C}\right)/\rho_{max}$ of the PdPC model close to bifurcation ($v_{2}=2.47$) as resulted from (**a**) numerical integration of the master equation via Runge–Kutta method ($\rho_{max}=9.32\times 10^{-4}$); (**b**) Random Walk Approximation ($\rho_{max}=7.24\times 10^{-4}$); (**c**) System Size Expansion ($\rho_{max}=9.11\times 10^{-4}$) and (**d**) linear approximation of the master equation (multinomial distribution) ($\rho_{max}=5.69\times 10^{-3}$). N=205, $k_{1}=0.1,k_{2}=1$ for all plots.

A visual picture of the results is given in Figure 4 and Figure 5, for the monostable and the bistable regime respectively, while the distributions close to criticality are shown in Figure A3. The numerical solution is shown in plot (**a**) of Figure 4 and Figure 5. We can observe that the SSE (plots (**c**) of Figure 4 and Figure 5) has good local performances around the critical point but fails in capturing the tails, which are better approximated by the global RWA (plot (**b**) of Figure 4 and Figure 5). In Figure 5, we notice that if the mean of the Gaussian is close to the boundary the SSE is poorer, in that the Gaussians are cut, resulting in a distortion of the final probability. This is likely why the SSE performs worse than the RWA close to the bifurcation and after, since the numerical distribution flattens and reaches the boundaries of the state space. This also explains why the SSE does not decrease with *N* in plots (**b**) and (**c**) of Figure 3 as expected, since it is a N→∞ approximation. On the other hand, the multinomial solution (plots (**d**) of Figure 4 and Figure 5) is always much worse than the other two, being able to capture only a very narrow neighborhood around the critical point. We remark that for all of them, the mode was the correct one, i.e., $x^{*}$, so the differences between the distributions were given by larger order moments.

The RWA has also the advantage that, being global, it allows an estimate of the transition rate between the two states, because it gives the energy barrier between the two stable states, which is in turn proportional to the Fiedler’s eigenvalue. Approximating the energy barrier is useful for the application of Kramers theory of transition rates.

Finally, we note that the numerical RK solution was compared with the solution obtained by means of the Gillespie algorithm [18] to ensure that the numerical integration was arrived at convergence to the stationary state. The results were exactly the same either considering the numerical integration or the Gillespie Monte Carlo simulation as benchmark, and therefore, the RK integrated solution can be considered as the “true” solution of the ME.

## 6. Discussion

We presented a new way to approximate the stationary distribution of stochastic processes governed by an ME, in general out of equilibrium (since we did not impose detailed balance), that can be mapped on a graph. We called this method the Random Walk Approximation, since it is based on the properties of the Laplacian matrix of random walk dynamics on graphs, which has been already successfully applied for instance for graph clustering [33,34]. Here, Laplacian theory on graphs is applied to justify the genesis of the RWA and in general to understand its range of applicability. The goal of the approximation is to give a stationary solution at the mesoscale, where stochasticity is important and the system is not at the thermodynamic limit (N→∞).

Summarizing, the essence of the RWA is to transform the complex problem of solving a non-linear ME (for the linear ME the analytical result and the RWA coincide) into an eigenvalue problem. Indeed, once the state-dependent rate matrix is given, all that is needed is to compute the null eigenvector of the Laplacian matrix associated to the process. We emphasize that this needs to be performed only once for each model, since the eigenvector will be parametrically dependent, and changing the parameters value can be easily achieved by replacement, while this is not the case for the numerical solutions of the ME. Therefore, the RWA can be preferable to the other techniques if one wants to make a systematic parametric study, exploring a large set of parameter values. Then, the eigenvector can easily be injected in formula (8) and computed for every point of the state space with any standard computational software, giving the stationary probability distribution.

Notably, the error of the RWA is related to the norm of the Jacobian matrix associated to the transition rates as a function of the local density. Therefore, the RWA is valid when we have an adiabatic variation of the transition rates in the state space. The RWA procedure can be applied in principle for any possible number of system states *M*, regardless of the number of particles *N* (even though for very large *N*, a deterministic approach may be often preferable), but both the complexity of the eigenvalue problem (which is of order $O(M^{3})$ [35]) and the product in Equation (8) become numerically prohibitive for large *M*. However, it has to be said that in those cases, also the other methods, including the numerical ones, are computationally very expensive, and an ME approach is often impossible.

Although the RWA is computationally faster than both direct RK integration and the Gillespie algorithm, if one needs a very accurate prediction of the stationary distribution, those numerical methods are still in most cases preferred. The main added value of our approximation is to have a simple global analytical form that can be used for further computations on the stochastic system, such as entropy production, or to explore the importance of rare mesoscale states contained on the tail of the distribution. This is something that a numerical solution does not easily allow and something that, even in the simpler monostable regime, with the SSE is quite complicated to achieve, involving many steps, both numerical and analytical, as described in Section 3.1. Moreover, the RWA does not decrease its accuracy when the probability distribution is close to the boundaries, since all the constraints are automatically included in the procedure.

We also showed that the RWA has the advantage of including the bifurcation in itself, meaning that the multi-stability does not need to be added artificially as for the SSE, and therefore, an a priori stable solution of the deterministic dynamics is not needed. Nevertheless, as a drawback, the RWA is mathematically reliable only before the bifurcation, when one stable solution exists. Although this may be an issue in more complex models, at least for the systems that we studied in this paper, the RWA performs well even during and after the bifurcation, setting the foundation to further studies that may make our results more general, at least under some conditions.

Finally, with respect to the SSE, our approximation is simpler, global and has comparable, or better, accuracy, when the thermodynamic limit is not verified and the number of particles is not very large (of the order of Avogadro number).

## Figures and Tables

**Figure A2:**
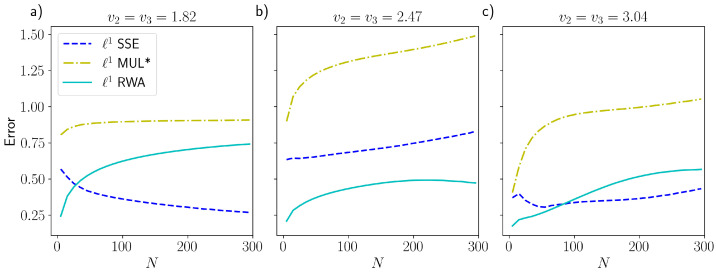
$\ell^{1}$-error with respect to the Runge–Kutta numerically integrated distribution vs. the number of particles *N* for the PdPC model. The colors refer to multinomial approximation (dashed–dotted yellow, referred to as MUL${}^{*}$), System Size Expansion (dashed blues) and Random Walk Approximation (solid cyan): (**a**) Monostable state with control parameter $v_{2}=1.82$. (**b**) Close to criticality with $v_{2}=2.47$. (**c**) Bistable with $v_{2}=3.04$. The other parameters are set to $k_{1}=0.1,k_{2}=1$ for all plots.

## Data Availability

All the code used for the simulations and relative data are available upon request to the corresponding author and on GitHub at https://github.com/tommasomarzi/random-walk-approximation (accessed on 17 February 2023).

## Abbreviations

The following abbreviations are used in this manuscript:

ME	Master Equation
RWA	Random Walk Approximation
SSE	System Size Expansion
RK	Runge–Kutta
FP	Fokker–Planck
PdPC	phospho/dephosphorylation cycle
JS	Jensen–Shannon divergence

## Appendix A. Entropic Derivation of the Multinomial Solution

We show here that the distribution (3) can be derived from an entropic principle assuming the statistical weight

$$w\left(\vec{n}\right)=\frac{N!}{n_{1}!\ldots n_{M}!}$$

for any physical state of the network $|\vec{n}|=N$, regardless of whether the Laplacian *L* satisfies the detailed balance condition. Since the particles are identical and $w(\vec{n})$ counts in how many ways the state $\vec{n}$ can be realized by *N* particles, the ratio $\rho \left(\vec{n}\right)/w\left(\vec{n}\right)$ is the statistical weight of the microstate. Using the Lagrangian multipliers to consider the constraints on the average number of particles for each node, we obtain the variational principle

$$\delta F\left[\rho \right]=-\sum_{|\vec{n}|=N}\delta \left[\rho \left(\vec{n}\right)\log\left(\frac{\rho (\vec{n})}{w(\vec{n})}\right)\right]+\sum_{i}\mu_{i}\sum_{|\vec{n}|=N}\delta \left[n_{i}\rho \left(\vec{n}\right)\right]=0.$$

Therefore, the stationary solution of (1) is

(A1) $$\rho \left(\vec{n}\right)\propto w\left(\vec{n}\right)\prod_{i}e^{\mu_{i}n_{i}}$$

and the choice $\mu_{i}=\log\left(p_{i}\right)$ provides $\langle n_{i}\rangle =Np_{i}$.

## Appendix B. Scaling of the Normalization Factor of the RWA

In order to show that C(N) does not have a strong dependence on *N*, we consider small perturbations of $\vec{p}\left(\vec{x}\right)$ around the critical points: $p_{k}\left(\vec{x}\right)=p_{k}^{*}+\Delta p_{k}\left(\vec{x}\right)$ where the perturbations $\Delta p_{k}$ sqatisfy the condition:

$$\sum_{k}\Delta p_{k}=0.$$

Then,

$$\sum_{|\vec{n}|=N}N!\prod_{k}\frac{p_{k}^{n_{k}}\left(\vec{x}\right)}{n_{k}!}=\sum_{|\vec{n}|=N}N!\prod_{k}\frac{{p_{k}^{*}}^{n_{k}}}{n_{k}!}{\left(1+\frac{\Delta p_{k}\left(\vec{x}\right)}{p_{k}^{*}}\right)}^{n_{k}}$$

and for N≫1, we obtain

$$C^{-1}\left(N\right)≃\sum_{|\vec{n}|=N}N!\prod_{k}\frac{{p_{k}^{*}}^{n_{k}}}{n_{k}!}\exp\left(\sum_{k}\frac{n_{k}\Delta p_{k}\left(\vec{x}\right)}{p_{k}^{*}}\right)$$

The main contributions to the probability distribution are when $|n_{k}-Np_{k}^{*}|\leq O\left(N^{1/2}\right)p_{k}^{*}$, according to the law of large numbers, and we obtain:

$$C^{-1}\left(N\right)≃\sum_{|\vec{n}|=N}N!\prod_{k}\frac{{p_{k}^{*}}^{n_{k}}}{n_{k}!}\exp\left(\sum_{k}\frac{\Delta p_{k}\left(\vec{x}\right)}{p_{k}^{*}}O\left(N^{1/2}\right)\right)$$

so that if $\Delta p_{k}$ is $O(N^{-1/2})$, we have the estimate C(N)=O(1) with a weak dependence on *N* and $p_{k}^{*}$.

## Appendix C. Error of the RWA

Starting from Equation (10), we apply the fact that if $n_{j}=Np_{j}\left(\vec{x}\right)=Nx_{j}$, the expression vanishes; then, one can consider only the contribution of the fluctuations, which are expected of order $O(\sqrt{N})$, according to the law of large number. Indeed, an explicit calculation gives:

$$\sum_{i,j}\pi_{ij}\left(\vec{x}\right)p_{j}\left(\vec{x}\right)\left(\frac{\partial p_{k}}{\partial x_{j}}-\frac{\partial p_{k}}{\partial x_{i}}\right)\frac{n_{k}}{p_{k}}=\sum_{i,j}\left(\pi_{ij}\left(\vec{x}\right)p_{j}\left(\vec{x}\right)\frac{\partial p_{k}}{\partial x_{j}}-\pi_{ji}\left(\vec{x}\right)p_{i}\left(\vec{x}\right)\frac{\partial p_{k}}{\partial x_{j}}\right)\frac{n_{k}}{p_{k}}.$$

Then, using the local stationarity condition

$$\sum_{i}\pi_{ji}\left(\vec{x}\right)p_{i}\left(\vec{x}\right)=\sum_{i}\pi_{ij}\left(\vec{x}\right)p_{j}\left(\vec{x}\right),$$

the previous expression vanishes.

Therefore, the main contribution to the error estimated from (10) is:

$$\frac{1}{N}\sum_{i,j}\pi_{ij}\left(\vec{x}\right)\Delta n_{j}\sum_{k}\rho_{\vec{p}}^{*}\left(\vec{n}\right)\left(\frac{\partial p_{k}}{\partial x_{j}}-\frac{\partial p_{k}}{\partial x_{i}}\right)\frac{\Delta n_{k}}{p_{k}}=O\left(∥\frac{\partial p}{\partial x}∥\right)$$

since the fluctuations are of order $\Delta n_{i}=p_{i}O\left(\sqrt{N}\right)$. The spectral properties of the Laplacian operator (2) follow from the ones of the transition rate matrix, and the Fiedler’s eigenvalue is independent of *N*. Then, the estimate

$$\sum_{i,j}\left[E_{j}^{+}E_{i}^{-}\pi_{ij}\left(\vec{x}\right)n_{j}-\pi_{ji}\left(\vec{n}\right)n_{i}\right]\rho_{\vec{p}}^{*}\left(\vec{n}\right)=O\left(∥\frac{\partial p}{\partial x}∥\right)$$

implies that estimate for the $\ell^{1}$-error is Equation (11), which is given in the main text.

## Appendix D. Runge–Kutta Algorithm

Runge–Kutta (RK) methods [36] are a family of iterative algorithms which allow to approximate the solution of initial value problems through numerical integration. In particular, at each step, the solution of the ordinary differential equation is computed by adding to its current value a fixed number of weighted increments. The latter are given by the slope of the solution evaluated at different points of the time step, and the weights are computed according to the instant of evaluation. If we denote as *h* the step size, an RK method of $n^{th}$ order performs a local truncation error of order $O(h^{n+1})$, which leads to an accumulated (or global) error of order $O(h^{n})$. For example, the most well-known method belonging to this family is the RK4 method [37], which commits a global error of order $O(h^{4})$. In Section 5, we will use an adaptive version of RK4, namely the RK5(4) [25] (or Runge–Kutta–Dormand–Prince) method: at each step, the algorithm evaluates the solution in parallel through an RK4 and an RK5 method and computes the subsequent time increment as a function of the difference of these solutions. Since the implementation of two parallel algorithms is computationally demanding, the solutions are evaluated using the same increments, and the associated weights are chosen to minimize the RK5. The whole procedure results in a fourth-order method, and the adaptive step size extends the region of absolute stability of the algorithm.

Starting from the one-step process ME in Equation (6), we can systematically build a dynamical linear system which takes into account all the possible exchanges of particles [26]. First of all, we denote as P the M-dimensional normalized and ordered vector with components representing the probability associated to each possible state $\vec{n}$. In a closed system, the dimension M is given by:

(A2) $$M=\prod_{i=1}^{M}{\left(N_{i}+1\right)}^{M}$$

where $N_{i}$ is the maximum number of particles of the species *i*. By keeping the same indexing of P(t), we can properly build the M×M Laplacian matrix *G* of the stochastic process: each off-diagonal element represents the transition rate associated to the one-particle exchange evaluated on a specific state, while the diagonal elements normalized each column to zero (accordingly to the canonical definition of the Laplacian matrix in Equation (2)). Because of the properties of *G*, the one-step process ME can be represented in terms of the following autonomous and positive linear system:

(A3) $$\dot{P}=GP$$

A priori, starting from an initial condition, we can make the system relax toward the stationary solution by using a numerical integration algorithm such as the RK methods. However, as the number of species and particles increases, the number of equations M grows drastically, and computations may become impractical in terms of processing power and times of convergence of the algorithm. Possible dependencies between the species result in a reduction of the dimension of $\vec{n}$: the state of a system can be described by a vector with dimension $M-M_{c}$, where $M_{c}$ is the number of mass balance constraints. This also implies a reduction of the dimension of M, since the probability associated to states that are not allowed is set to zero. Furthermore, the one-step process hypothesis results in a high sparsity of *G*, since the only non-zero elements are those associated to the exchanges of one particle. Indeed, the structure of *G* is a block-tridiagonal matrix, leading to some numerical advantages [26]. The analysis was carried out by means of Python 3.10.4, mainly by means of module scipy v1.9.1, based on [24,25].

## Appendix E. System Size Expansion of the ME

Since we consider the one-step process evolution, in the limit N→∞, it is convenient to rescale the time unit Δt→Δt/N to obtain a finite relaxation time, as in Section 2. Then, the ME (1) can be written in the form

$$\begin{array}{ccc} & & \frac{\partial \rho }{\partial t}\left(\vec{x},t\right)=\sum_{i,j}\left[E_{j}^{+}E_{i}^{-}\pi_{ij}x_{j}-\pi_{ji}x_{i}\right]\rho \left(\vec{x},t\right)\\ & & ≃\sum_{i,j}\left(\frac{\partial }{\partial x_{j}}-\frac{\partial }{\partial x_{i}}\right)\pi_{ij}x_{j}\rho \left(\vec{x},t\right)+\frac{1}{2N}\sum_{i,j}{\left(\frac{\partial }{\partial x_{j}}-\frac{\partial }{\partial x_{i}}\right)}^{2}\pi_{ij}x_{j}\rho \left(\vec{x},t\right) \end{array}$$

up to terms of order $O(N^{-2})$. If one approximates the distribution in a neighborhood $\delta \vec{x}=\vec{x}-{\vec{x}}^{*}$ of the mode value ${\vec{x}}^{*}$, we obtain the Fokker–Planck (FP) equation for the SSE:

(A4) $$\begin{array}{ccc} \frac{\partial \rho }{\partial t}\left(\delta \vec{x},t\right) & = & \sum_{i,j}\frac{\partial }{\partial x_{j}}\left(\pi_{ij}x_{j}-\pi_{ji}x_{i}\right)\rho \left(\delta \vec{x},t\right)\\ & + & \frac{1}{2N}\sum_{i,j}\left(\pi_{ij}x_{j}^{*}+\pi_{ji}x_{i}^{*}\right)\left(\frac{\partial^{2}}{\partial x_{i}^{2}}-\frac{\partial^{2}}{\partial x_{i}\partial x_{j}}\right)\rho \left(\delta \vec{x},t\right). \end{array}$$

The drift term is a linear force field associated to the Laplacian matrix $L_{ij}$, whereas the diffusion coefficient is defined by the positive symmetric matrix

$$D_{ij}=\frac{1}{N}\left[\sum_{k}\left(\pi_{ik}x_{k}^{*}+\pi_{ki}x_{i}^{*}\right)\delta_{ij}-\left(\pi_{ij}x_{j}^{*}+\pi_{ji}x_{i}^{*}\right)\right]=\frac{1}{N}\left[L_{ij}x_{j}^{*}+L_{ji}x_{i}^{*}\right]$$

The stationary distribution of the FP equation (A4) is a Gaussian function [8], with average value $x_{i}=x_{i}^{*}$ and covariance matrix *C* that satisfies the Lyapunov equation

(A5) $$\dot{C}=-LC-CL^{T}+D$$

Then, the covariance matrix for the stationary distribution satisfies the equation

$$L_{ik}C_{kj}+C_{ik}L_{kj}^{T}+\frac{1}{N}\left(L_{ij}p_{j}+L_{ji}p_{i}\right)=0$$

and by a direct calculation, we obtain a solution of the form

$$C_{ij}=\frac{1}{N}\left(p_{i}\delta_{ij}-p_{i}p_{j}\right)$$

that coincides with (4).

The SSE is an approximation of the multinomial distribution (3) as N≫1. In general, for non-linear master equations, the FP reads:

(A6) $$\frac{\partial \rho (\vec{x},t)}{\partial t}=\sum_{i}\frac{\partial }{\partial x_{i}}A_{i}\left(\vec{x}\right)\rho \left(\vec{x},t\right)+\frac{1}{2}\sum_{i,j}\frac{\partial^{2}}{\partial x_{j}\partial x_{i}}D_{ij}\left(\vec{x}\right)\rho \left(\vec{x},t\right)$$

in which we defined the drift term and the diffusion term, respectively, as:

$$\begin{array}{cc} A_{i}\left(\vec{x}\right) & =\sum_{j}\left(\pi_{ji}\left(\vec{x}\right)x_{i}-\pi_{ij}\left(\vec{x}\right)x_{j}\right)=\sum_{j}L_{ij}\left(\vec{x}\right)x_{j}\\ D_{ij}\left(\vec{x}\right) & =\frac{1}{N}\left[\sum_{k}\delta_{ij}\left(\pi_{ik}\left(\vec{x}\right)x_{k}+\pi_{ki}\left(\vec{x}\right)x_{i}\right)-\left(\pi_{ij}\left(\vec{x}\right)x_{j}+\pi_{ji}\left(\vec{x}\right)x_{i}\right)\right] \end{array}$$

The System Size Expansion is the key step to obtain a linear FP equation. In particular, it consists in assuming the linearity of this equation by requiring that the drift field is linear and the diffusion coefficient is constant with respect to $\vec{x}$. Since it allows approximating the distribution near the critical value, we develop the drift field near the vector ${\vec{x}}^{*}$ up to the first order, and we compute the diffusion coefficient in the same point. The linearized FP equation associated to the SSE reads:

(A7) $$\frac{\partial \rho (\delta \vec{x},t)}{\partial t}=\sum_{i}\frac{\partial }{\partial x_{i}}\left(\sum_{j}L_{ij}^{*}\delta x_{j}+\sum_{j,k}\frac{\partial L_{ij}}{\partial x_{k}^{*}}x_{j}^{*}\delta x_{k}\right)\rho \left(\delta \vec{x},t\right)+\frac{1}{2}\sum_{i,j}\frac{\partial^{2}}{\partial x_{j}\partial x_{i}}D_{ij}^{*}\rho \left(\delta \vec{x},t\right)$$

which holds in a neighborhood $\delta \vec{x}=\vec{x}-{\vec{x}}^{*}$ of the critical point ${\vec{x}}^{*}$. The solution of Equation (A7) is known again to be a normal distribution [8]. Its stationary mean and covariance can be obtained by imposing the time-derivative of the first and second moment equal to zero. Thus:

(A8) $$\frac{\partial \langle \delta x_{h}\rangle }{\partial t}=\int d\delta \vec{x}\;\delta x_{h}\;\frac{\partial \rho (\delta \vec{x},t)}{\partial t}$$

and

(A9) $$\frac{\partial \langle \delta x_{h}\delta x_{l}\rangle }{\partial t}=\int d\delta \vec{x}\;\delta x_{h}\delta x_{l}\;\frac{\partial \rho (\delta \vec{x},t)}{\partial t}.$$

From Equations (A8) and (A9), by injecting (A7) and integrating by parts, one can prove that the mean of the stationary distribution is centered in ${\vec{x}}^{*}$, while the covariance matrix Σ solves the continuous Lyapunov equation [8]:

(A10) $$-\bar{A}\Sigma -\Sigma {\bar{A}}^{T}+D^{*}=0$$

in which we denoted as $\bar{A}$ the matrix such that:

(A11) $${\bar{A}}_{ij}=\sum_{k}\left[L_{ik}^{*}\delta_{jk}+\frac{\partial L_{ik}}{\partial x_{j}^{*}}x_{k}^{*}\right].$$

Therefore, a local stationary solution which holds in a neighborhood of the critical point ${\vec{x}}^{*}$ is obtained by the Gaussian distribution given in the main text:

(A12) $$\rho_{SSE}^{*}\left(\delta \vec{x}\right)=\frac{1}{{\left(2\pi \right)}^{3/2}\;{\left|\Sigma \right|}^{1/2}}\exp\left[-\frac{1}{2}\delta {\vec{x}}^{T}\Sigma^{-1}\delta \vec{x}\right].$$

## Appendix F. Supplementary Results

In Figure A1a, we have computed the relative error Δσ between the variance σ of the numerical solution of the ME associated to Equation (15) for the model and the variance $\sigma_{m}$ computed by the RWA, which turns out to be an overestimate of the numerical variance

$$\Delta \sigma =\frac{\sigma_{m}-\sigma }{\sigma }.$$

We observe that the error Δσ is independent of the particle number, it is directly related to the $\ell^{1}$-error of the RWA, and the error increases abruptly near the bifurcation value α=0.8 with a dependence from *N*.

Figure A1c shows the $\ell^{1}$-error as a function of α, which is approximately linear for small α, far from the bifurcation, and increases monotonically for increasing values of α. At the bifurcation, there is the emergence of abrupt changes in the monotony of the function. Moreover, we note once again that far from the bifurcation, there is no dependence on *N*, as expected theoretically.
